# Digitising payments for campaign health workers in Africa: the promise and the path to sustainable scale

**DOI:** 10.1136/bmjgh-2025-022678

**Published:** 2026-02-15

**Authors:** Peter Waiswa, Juliet Aweko, Margaret McConnell, Ahmed Hamani, Oswell Kahonde, Eric Aigbogun, Chukwunonso Nwaokorie, Uchenna Igbokwe, Elizabeth Ekirapa Kiracho, Digital Health Payments Initiative and Research (DHPIR) Consortium, Peter Waiswa

**Affiliations:** 1 Department of Health Policy, Planning and Management, Makerere University School of Public Health, Kampala, Uganda; 2 Global Health and Population, Harvard T.H. Chan School of Public Health, Boston, Massachusetts, USA; 3 World Health Organization Regional Office for Africa, Brazzaville, Bahamas; 4 The Better than Cash Alliance, Kigali, Rwanda; 5 Anatomy, Enugu State University of Science and Technology College of Medicine, Abuja, Nigeria; 6 5Solina Centre for International Development and Research (SCIDaR), Abuja, Nigeria

**Keywords:** Poliomyelitis, Vaccines, Immunisation, Public Health, Child health

**Summary box**

- Front-line health workers are essential for mass vaccination campaigns in Africa, but their motivation and performance are often undermined by delayed, insecure and inefficient cash-based payment systems.
- Digital payment systems provide a faster, more secure and transparent mechanism for financial transactions. Evidence indicates that their adoption can significantly shorten payment processing times, from several weeks to a matter of hours, while enhancing traceability, reducing errors and improving overall accountability in fund disbursement.
- Evidence from the Digital Health Payment Initiative and Research consortium across 12 African countries confirms significant efficiency gains but also reveals critical systemic bottlenecks and gender-related barriers that hinder equitable scale-up.
- Scaling effectively goes beyond technology; it requires strategic investment in administrative systems, inclusive and gender-responsive design, and robust digital infrastructure.
- Integrating digital payments into national health programmes can enhance trust, equity and system resilience. A focused research agenda is essential to fully harness their public health potential.

## Introduction

 Large-scale vaccination and disease-control campaigns remain a cornerstone of public health, critical to combating infectious threats from polio and yellow fever to measles and cholera.^1^ Yet these campaigns now unfold in a sharply constrained funding environment, with global health resources under pressure even as outbreaks rise in frequency and scale. In this new era, Africa must do more with less: prevent diseases proactively, use domestic and donor resources more efficiently, and ensure every dollar spent produces maximum public health impact.^2^

At the heart of these efforts is the campaign health workforce: tens of thousands of community-based and temporary health workers dedicated to delivering essential services to some of the most remote and underserved populations. How health workers are paid directly influences their motivation, timeliness and trust, which are key drivers of campaign success.^3^ However, for decades, there has been heavy reliance on cash-based systems, which have caused delays in payment, eroded morale and undermined quality of health campaigns.^4^ In early 2020, slow distribution of funds disrupted or delayed around half of all polio outbreak campaigns in the WHO African region.^5 6^

The ongoing digital transformation across Africa, driven by mobile connectivity, fintech innovation and government ‘zero cash’ agendas, offers an unprecedented opportunity for change. Digital payments via mobile money piloted in Côte d'Ivoire, Democratic Republic of the Congo, Ghana, Mali and Republic of the Congo have proven to cut payment turnaround from weeks to hours, enhance accountability and improve payment security.^6^ In Côte d'Ivoire, for instance, payment times fell from 3 weeks to just 2 hours, ensuring over 50 000 workers were paid fully and on time.^6^

However, while pilots have shown promise, scaling up such systems across the continent has been hindered by a lack of robust, context-specific evidence. The Digital Health Payment Initiative and Research (DHPIR) consortium was created to close this gap, generating the first multicountry body of research on how digitising payments can support efficiency, motivation and equity. This inaugural supplement (https://gh.bmj.com/content/10/Suppl_4) on digital payments for campaign health workers, published in *BMJ Global Health*, consolidates evidence from across sub-Saharan Africa. It shows that timely, transparent digital payments go beyond administrative efficiency; they are essential to ensuring quality, motivation and fairness.

### The consensus research agenda: a framework for scale

We used the Child Health and Nutrition Research Initiative (CHNRI) process to understand the research priorities for scaling digital payments systems. Our CHNRI work engaged 150 stakeholders to rank the most critical unanswered questions.^7^ The top priority questions coalesced around five themes: (1) System Requirements, (2) Optimisation, (3) Incentives and Adoption, (4) Cost-Effectiveness and (5) Equity. These are not isolated topics but interconnected pillars for building effective digital payment ecosystems.

### System requirements: the foundational bottleneck

The top-ranked research question asks: What are the minimum requirements for health systems to digitise payments responsibly? This prioritisation reflects a hard truth revealed by DHPIR studies: technology fails without robust administrative foundations. For instance, a cluster randomised trial in Uganda found that even with digital systems, payments were still delayed by a median of 40 days postcampaign.^7^ The bottlenecks were not technological but systemic: incomplete worker registries, manual and protracted verification processes, and multilayered bureaucratic approvals.^7^ Similarly, a feasibility study of Uganda’s yellow fever campaign highlighted that success depended on pre-existing, robust payment platforms and dedicated verification teams.^8^ Research must, therefore, define the necessary preconditions for data systems, streamlined workflows and inter-ministerial coordination protocols to prevent digitisation from simply automating existing inefficiencies.

### Optimisation: from functional to effective

The second priority seeks strategies to enhance the effectiveness of digital systems in large-scale campaigns. Evidence confirms that while digital payments can be faster, their impact on ultimate campaign outcomes is not automatic. The Ugandan RCT found no significant difference in health worker motivation between digital and cash arms, largely due to the persistent delays common to both.^7^ This indicates that the mere presence of a digital channel is insufficient. Future research must investigate how to optimise these systems through better integration with campaign logistics, real-time payment tracking and dynamic feedback mechanisms to directly improve coverage, quality and worker performance.

### Incentives and adoption: engaging the ecosystem

Understanding barriers and enablers for government and institutional uptake is crucial for moving beyond donor-funded pilots. The research agenda signals that the ‘why’ of adoption is as important as the ‘how’. While digital payments offer efficiency, their implementation requires upfront investment and shifts in institutional culture and power dynamics. Studies suggest that government ownership and clear demonstration of value such as freeing district officials from cash management to focus on supervision^8^ are key incentives. Research must map the political economy of digitisation, identifying incentives for ministries of health, finance and telecommunications to collaborate and sustainably fund these systems.

### Cost-effectiveness: making the investment case

A rigorous evaluation of the long-term value and sustainability of digital vs cash systems is essential for policymakers allocating scarce resources. Pilots in Côte d’Ivoire show dramatic time savings,^5^ but a full accounting must include the costs of technology, agent networks, system maintenance and change management. Research must move beyond operational efficiency to measure broader value: reduced financial leakage, improved campaign effectiveness, higher worker retention and strengthened trust in the health system. This evidence is critical to justify the transition from pilots to integrated national systems.

### Equity: the imperative for gender-transformative design

Ensuring systems promote financial inclusion and do not leave women or marginalised groups behind emerged as a paramount concern, powerfully underscored by the supplement’s qualitative findings.^9^ A study in Uganda and Malawi revealed that digital payments are not inherently empowering. While they offered women health workers greater autonomy and time savings, structural barriers such as using a husband’s phone for registration often rerouted control of earnings to men. Delays or poor communication about payments sometimes triggered intimate partner violence and mistrust.^9^ This evidence dictates that research must investigate and promote gender-transformative design. This means moving beyond gender-blind systems to actively address norms, ensuring women have direct access to phones and accounts, and embedding safeguards against backlash. Equity-focused research is essential to prevent digitisation from deepening existing social inequities.

### A call to action: from agenda to evidence

The DHPIR consortium has moved the field from documenting pilot outcomes to defining a structured pathway for inquiry. This research agenda provides a critical tool for alignment. We call on funders, research institutions and policymakers to direct resources towards these five priority domains.

Scaling digital payments is not merely a technical upgrade but a systemic reform that touches on governance, finance and social justice. By investing in this agenda, we can generate the evidence needed to build digital payment systems that are not only efficient but also equitable, sustainable and effective, ensuring that every health worker is paid with the dignity and timeliness they deserve, and that every campaign achieves its life-saving potential.

## Supplementary material

10.1136/bmjgh-2025-022678 online supplemental file 1

## Data Availability

No data are available.
