# Supplementary material for: Digitising payments for campaign health workers in Africa: the promise and the path to sustainable scale
Source: BMJ Glob Health. 2026 Feb 15;10(Suppl 4):e022678. doi: 10.1136/bmjgh-2025-022678 (PMC12962002; doi:10.1136/bmjgh-2025-022678)
Supplement: online supplemental file 1 [file bmjgh-10-Suppl_4-s001.docx]

### BMJ Global Health Author Reflexivity Statement

Adapted from Morton, B., Vercueil, A., Masekela, R., Heinz, E., Reimer, L., Saleh, S., Kalinga, C., Seekles, M., Biccard, B., Chakaya, J., Abimbola, S., Obasi, A. and Oriyo, N. (2022), Consensus statement on measures to promote equitable authorship in the publication of research from international partnerships. Anaesthesia, 77: 264-276. <https://doi.org/10.1111/anae.15597>

| **Study conceptualisation** | |
| --- | --- |
| 1. How does this study address local research and policy priorities? | Improving the efficiency and equity of payments for frontline health workers involved in large scale vaccination campaigns is critical for the success of mass vaccination campaigns. Recommendations from this paper lay a foundation for the policy dialogues on scalability of digital payments across sectors. It also highlights key research questions from the research agenda to be prioritized for further research on digital payments. |
| 1. How were local researchers involved in study design? | Local researchers from institutions in Uganda (Makerere University) and Nigeria (SCIDaR) are listed as lead and co-authors, suggesting central involvement. The multi-country, consortium-based approach (DHPIR) employed a collaborative design. |
| **Research management** | |
| 1. How has funding been used to support the local research team(s)? | Funding from the Bill & Melinda Gates Foundation supported the work and the supplement, which inherently includes supporting the local research teams at the African partner institutions for their time, data collection, and analysis. |
| **Data acquisition and analysis** | |
| 1. How are research staff who conducted data collection acknowledged? | While specific data collectors are not named, the authors acknowledge "all research participants and partners," which typically includes field staff. |
| 1. How have members of the research partnership been provided with access to study data? | There is no data available for this commentary as it discusses high-level findings from the multi-country studies conducted through collaborative partnership in the digital health payment project. |
| 1. How were data used to develop analytical skills within the partnership? | The application of multiple analytical approaches including; statistical and qualitative techniques to interpret data contributed to the validity and reliability of the research findings and supported researchers make evidence-based conclusions and recommendations. |
| **Data interpretation** | |
| 1. How have research partners collaborated in interpreting study data? | Through a iterative process of synthesizing evidence from the twelve countries and the development of a unified conceptual model and collective development of the research agenda strongly indicates that research partners collaborated deeply in interpreting the data across different contexts. |
| **Drafting and revising for intellectual content** | |
| 1. How were research partners supported to develop writing skills? | The research partners were mentored through hybrid writing workshops to support the teams build their writing skills. |
| 1. How will research products be shared to address local needs? | The research products will be disseminated through a virtual global webinar and the launch of the supplement which is to be published in high impact factor journal (BMJGH). |
| **Authorship** | |
| 1. How is the leadership, contribution and ownership of this work by LMIC researchers recognised within the authorship? | LMIC researchers hold the first, second, corresponding, and several senior author positions. The consortium is named after the lead author (P. Waiswa) and corresponding author (J. Aweko), both from Makerere University, Uganda. |
| 1. How have early career researchers across the partnership been included within the authorship team? | The early career researchers were strongly involved in the conceptualization, data collection, analysis and drafting of this manuscript. They hold the position of second (J. Aweko), third (O. Charles and fourth (AS. Maggie) authors in the manuscript |
| 1. How has gender balance been addressed within the authorship? | The authorship list shows a strong gender balance, with multiple women in prominent positions, including the corresponding author and the last author. |
| **Training** | |
| 1. How has the project contributed to training of LMIC researchers? | All the project studies were conducted in LMIC countries by local researchers from institutions in LMIC including; Makerere University, University of Dakar Senegal, Compelling works, SCIDAR which are all African. This has increased learning across contexts particularly between the Anglo and Francophone countries. |
| **Infrastructure** | |
| 1. How has the project contributed to improvements in local infrastructure? | The research directly studies and advocates for the improvement of digital payment infrastructure. While not explicitly stated, the partnerships and pilot programs likely contributed to strengthening local research and digital health systems. |
| **Governance** | |
| 1. What safeguarding procedures were used to protect local study participants and researchers? | This commentary did not require patient consent for publication as it presents arguments based on synthesized findings from studies with standard ethical approvals. |
